# Quantitative SPECT/CT Parameters in the Assessment of Transthyretin Cardiac Amyloidosis—A New Dimension of Molecular Imaging

**DOI:** 10.3390/jcdd10060242

**Published:** 2023-05-31

**Authors:** Mirela Gherghe, Alexandra Maria Lazar, Maria-Carla Sterea, Paula Monica Spiridon, Natalia Motas, Laurentia Nicoleta Gales, Daniel Coriu, Sorina Nicoleta Badelita, Mario-Demian Mutuleanu

**Affiliations:** 1 Nuclear Medicine Department, University of Medicine and Pharmacy “Carol Davila”, 050474 Bucharest, Romaniamario-demian.mutuleanu@drd.umfcd.ro (M.-D.M.); 2 Nuclear Medicine Department, Institute of Oncology “Prof. Dr. Alexandru Trestioreanu”, 022328 Bucharest, Romania; 3 Carcinogenesis and Molecular Biology Department, Institute of Oncology “Prof. Dr. Alexandru Trestioreanu”, 022328 Bucharest, Romania; 4 Department of Thoracic Surgery, University of Medicine and Pharmacy “Carol Davila”, 050474 Bucharest, Romania; 5 Clinic of Thoracic Surgery, Institute of Oncology “Prof. Dr. Alexandru Trestioreanu”, 022328 Bucharest, Romania; 6 Oncology Department, University of Medicine and Pharmacy “Carol Davila” Bucharest, 050474 Bucharest, Romania; 7 Oncology Department, Institute of Oncology “Prof. Dr. Alexandru Trestioreanu”, 022328 Bucharest, Romania; 8 Hematology Department, University of Medicine and Pharmacy “Carol Davila”, 050474 Bucharest, Romania; 9 Hematology Department, Fundeni Clinical Institute, 022322 Bucharest, Romania

**Keywords:** cardiac amyloidosis, ATTR amyloidosis, SPECT/CT, molecular imaging, amyloid burden, myocardium, restrictive cardiomyopathy

## Abstract

Aims: Cardiac transthyretin amyloidosis (ATTR) represents the accumulation of misfolded transthyretin in the heart interstitium. Planar scintigraphy with bone-seeking tracers has long been established as one of the three main steps in the non-invasive diagnosis of ATTR, but lately, single-photon emission computed tomography (SPECT) has gained wide recognition for its abilities to exclude false positive results and offer a possibility for amyloid burden quantitation. We performed a systematic review of the existing literature to provide an overview of the available SPECT-based parameters and their diagnostic performances in the assessment of cardiac ATTR. Methods and Methods: Among the 43 papers initially identified, 27 articles were screened for eligibility and 10 met the inclusion criteria. We summarised the available literature based on radiotracer, SPECT acquisition protocol, analysed parameters and their correlation to planar semi-quantitative indices. Results: Ten articles provided accurate details about SPECT-derived parameters in cardiac ATTR and their diagnostic potential. Five studies performed phantom studies for accurate calibration of the gamma cameras. All papers described good correlation of quantitative parameters to the Perugini grading system. Conclusions: Despite little published literature on quantitative SPECT in the assessment of cardiac ATTR, this method offers good prospects in the appraisal of cardiac amyloid burden and treatment monitoring.

## 1. Introduction

Amyloidosis represents a rare systemic disease characterised by protein misfolding and deposition, which usually leads to progressive organ failure [1,2,3]. Cardiac amyloidosis is highlighted by the extracellular deposition of the misfolded proteins in the heart, which can be demonstrated by a pathognomonic histological property after Congo red staining; namely, green birefringence under polarized light [2,3]. This infiltration of the heart interstitium by amyloid causes the stiffening of the myocardium, resulting in progressive cardiac dysfunction, which has a great impact on the quality of life [4]. Deposits can derive from different precursors, the most common accumulations being immunoglobulin-derived amyloid light chains (AL), produced by monoclonal plasma cells, and transthyretin amyloidosis (ATTR), which results from misfolded proteins synthesized by the liver [5,6]. At present, over 98% of diagnosed cardiac amyloidosis results from fibrils pertaining to one of the two aforementioned types [2].

AL and ATTR amyloidosis exhibit different and diverse patterns of deposition throughout the body, distinct clinical courses, dissimilar prognoses and specially tailored therapies [1,5,7]. Due to these distinct characteristics, AL and ATTR cardiac amyloidosis present differently in diagnostic tests. The gold standard for diagnosing cardiac amyloidosis is represented by endomyocardial biopsy with pathologic examination, in which amyloid fibrils are analysed and differentiated by mass spectrometry or immunofluorescence [8]. However, the invasiveness and scarce availability of this procedure has raised the need for non-invasive and broadly accessible diagnostic methods. Non-invasive diagnosis of cardiac amyloidosis is based on a multimodality approach, including imaging examinations and genetic and laboratory testing [4,9,10,11].

Cardiac amyloidosis can be suspected on conventional imaging studies, such as echocardiography and cardiac magnetic resonance (CMR), which offer important hints that can further guide the diagnostic process [4,12,13]. Echocardiography can exclude other causes of heart failure and left ventricular wall thickening; however, it cannot distinguish between the subtypes [14]. CMR is more effective than ultrasound in cardiac amyloidosis, as it conveys valuable information regarding tissue composition, revealing important parameters such as late gadolinium enhancement (LGE) and the extracellular volume (ECV) [15,16]. However, all the CMR findings are not specific to cardiac amyloidosis and may be observed in other heart diseases, such as sarcoidosis, infarction or myocardial injuries [12,15].

In comparison to other imaging investigations, molecular imaging, namely planar bone scintigraphy, together with single photon emission computed tomography (SPECT) and positron emission tomography (PET), plays a critical role in the diagnosis, identification and distinction between ATTR and AL cardiac amyloidosis [17]. There are several radiotracers that can investigate the presence of amyloid. ^123^I-labelled serum amyloid P component (^123^I-SAP) binds to all types of amyloid fibrils and it is usually used to establish the extent of systemic amyloidosis [18]; however, it has a low capability of detecting cardiac involvement, as ^123^I-SAP has a decreased permeability in heart tissue, and challenges in standardization and regulation raised difficulties in its worldwide use [19]. It has been known for over 40 years that bone-avid radiotracers, such as ^99m^Tc-labelled pyrophosphate (PYP), 3,3-diphosphono-1,2-propanodicarboxylic acid (DPD) and hydroxymethylene diphosphonate (HMDP), bind to amyloid deposits [20,21]. Recently, PET radiopharmaceuticals have been demonstrated to detect cardiac amyloid deposits and potentially to distinguish between the AL and the ATTR forms, but more data is needed to define their overall diagnostic accuracy [17,22]. Radiotracers such as ^11^C-Pittsburg compound B (PIB), and ^18^F-labelled agents (^18^F-florbetapir and ^18^F-florbetaben) are the two most common classes studied for this purpose [22,23,24].

**Figure 1:**
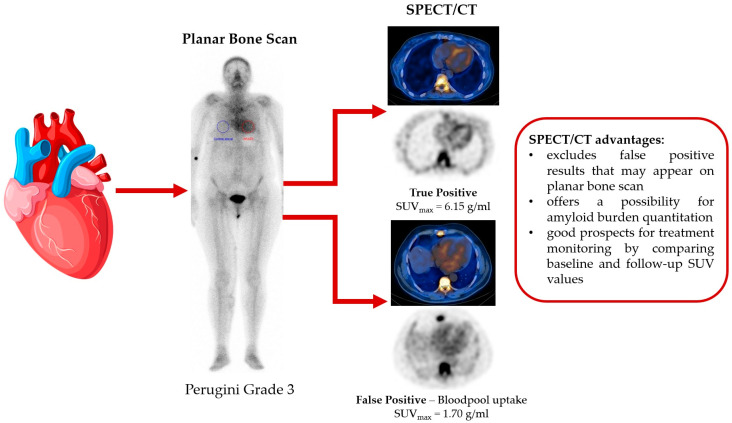
SPECT/CT advantages in comparison to planar scintigraphy in the assessment of cardiac transthyretin amyloidosis.

Although cardiac scintigraphy with bone-seeking tracers lacks the ability to offer structural or hemodynamic information, it can provide incremental value through its unique ability to differentiate between ATTR and AL amyloidosis [5,21,25]. Perugini et al. [26] were the first group of authors to establish a semiquantitative approach in differentiation between AL and ATTR amyloidosis based on planar ^99m^Tc-DPD scintigraphy. However, the disadvantages of this technique are the operator-dependence and its reliance on extracardiac sites as comparators, which can represent a problem in monitoring patients when abnormal extracardiac uptake is present [27]. Absolute and objective myocardial quantification might represent a method to overcome these shortcomings (Figure 1), especially in the case of patients who showcase lower grades of myocardial uptake (grades 1–2). Recent developments in SPECT gamma cameras, well as standardization of gamma camera calibration, have led to research into the possibility of utilising SPECT quantification in clinical practice [28]. Quantitative SPECT has been proven useful in the assessment of bone and neuroendocrine tumours [29,30,31]. Our review provides a qualitative report of the existing literature, describing the SPECT/CT quantitative methods used to assess the amyloid burden in patients with ATTR cardiac amyloidosis.

## 2. Materials and Methods

### 2.1. Aims of Review

Our review aims to describe the correlation between the quantitative parameters obtained on SPECT/CT with ^99m^Tc-labelled bone-seeking radiotracers and the semiquantitative indices derived from planar scintigraphy, with the objective of identifying the best criteria for diagnosing and monitoring treatment response in patients diagnosed with ATTR cardiac amyloidosis.

### 2.2. Search Algorithm

**Figure 2:**
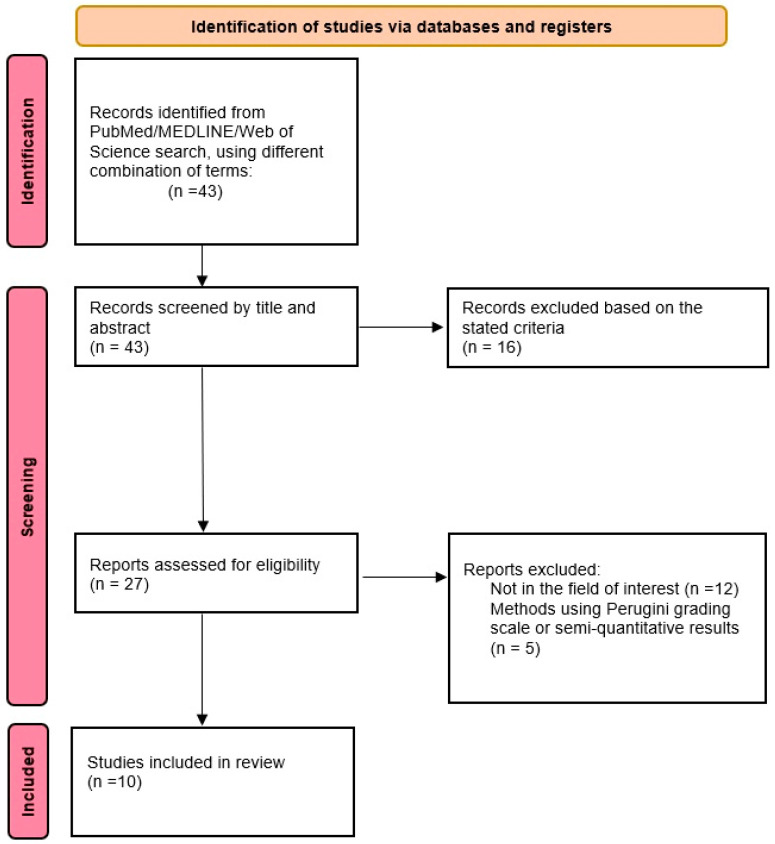
Schematic representation of the process of literature selection for this qualitative review.

A comprehensive search algorithm in the PubMed, MEDLINE and Web of Science databases was constructed based on the combination of the following terms: “transthyretin cardiac amyloidosis”, “SPECT/CT” and “quantitative SPECT/CT”. No beginning date was applied and the search extended up to January 2023. To expand our research, we manually evaluated references from the retrieved articles to search for supplementary useful studies. We followed the Preferred Reporting Items for Systematic Reviews and Meta-Analysis (PRISMA) guidelines to select the relevant studies [32] (Figure 2). All studies or subsets in studies investigating the quantification of myocardial ^99m^Tc-labeled radiotracer uptake in patients with transthyretin-related cardiac amyloidosis were considered eligible for inclusion. After removing duplicates, three authors (A.M.L., M.C.S. and P.M.S.) performed an initial screening of the identified titles and abstracts.

The inclusion criteria required: (a) articles written in English, (b) investigating patients suspected or diagnosed with transthyretin cardiac amyloidosis, (c) using ^99m^Tc-labelled bone-avid radiotracers for performing the investigation and (d) applying quantitative analysis based on standardized uptake values (SUV) derived from SPECT/CT images. Both prospective and retrospective studies were considered eligible.

The exclusion criteria were as follows: (a) articles not within the field of interest; (b) articles written in languages other than English; (c) case reports or small case series; (d) in vitro or animal studies; (e) reviews and meta-analysis articles, letters, comments, or conference proceedings.

### 2.3. Data Extraction and Synthesis

Among the 43 articles identified after the first search, 27 studies were assessed for eligibility, and 10 studies were selected and used for this qualitative synthesis. Data extracted from each publication included authors, research design, study reasons, imaging techniques, number of patients studied, type of cardiac amyloidosis, semiquantitative indices analysed on planar scintigraphy, investigated quantitative SPECT/CT indices and study results. A descriptive overview was performed to provide key summary statistics of the information obtained from the papers.

## 3. Results

Table 1 summarizes the main characteristics of the studies focused on quantitative SPECT/CT parameters in the assessment of ATTR cardiac amyloidosis.

The first study to analyse the potential utility of SPECT/CT quantitative parameters in assessing the ATTR cardiac amyloid was developed in 2018 by Ramsey et al. [33]. The authors investigated the concept of establishing a reference interval for ^99m^Tc-hydroxy-methylene diphosphonate (^99m^Tc-HDP) myocardial uptake that could distinguish the individuals with cardiac ATTR from those that were healthy or suffering from other types of cardiac amyloidosis. After separating 29 patients into four groups (group 1: ATTR cardiac amyloidosis; group 2: AL cardiac amyloidosis; group 3: other cardiac disease; group 4: non-cardiac patients), they discovered that both the semiquantitative and the quantitative indices were different in group 1 than in groups 2–4. They also stated that the SUV_max_ in groups 2–4 were similar, at the low end of the whole heart measurements, while the SUV_max_ values in the ATTR group were significantly higher. The authors concluded that a SUV_max_ cutoff of 1.2 would accurately distinguish between the ATTR-positive patients and the non-ATTR ones.

Caobelli et al. [34] tested the feasibility of quantitative ^99m^Tc-3,3-diphosphono-1,2-propanodicarboxylic acid (^99m^Tc-DPD) SPECT/CT parameters and their correlation to the Perugini score in 13 patients. They measured myocardial SUV_max_ and SUV_peak_ and also normalised their values to bone activity (nSUV_max_, nSUV_peak_). The authors discovered that both SUV_max_ and SUV_peak_ had a fairly strong linear relationship with Perugini score (*p* = 0.006), and the same was true for nSUV_max_ and nSUV_peak_ (*p* = 0.003). They also stated that a value of SUV_max_ > 3.3, respectively SUV_peak_ > 3.1, can reliably distinguish Perugini 2–3 patients from Perugini 0–1 individuals.

A larger number of SPECT-based parameters were evaluated by Bellevre et al. [35], in their paper that examined the feasibility of assessing absolute myocardial ^99m^Tc-hydroxy-dimethylene diphosphonate (^99m^Tc-HDMP) uptake in patients with suspected ATTR using a cadmium-zinc-telluride (CZT) SPECT/CT camera. The authors appraised the diagnostic potential of SUV_max_, percentage of injected dose (ID), heart-to-lung ratio (HLR) and muscle-to-bone ratio (MBR). They discovered that SUV_max_ and ID showed the best diagnostic accuracy in amyloidosis, with an area under the curve (AUC) of 1 for both, establishing that a threshold of 5.9 for SUV_max_ and 0.3 for ID had sensitivity and specificity of 100%.

Scully et al. [36] investigated whether SPECT/CT-derived SUV quantification could improve the diagnostic accuracy of ^99m^Tc-DPD scintigraphy, by correlating the planar scintigraphy indices with SUV_peak_ and cardiac SUV retention index values obtained on SPECT. They discovered that cardiac SUV_peak_ had a high diagnostic accuracy (AUC = 0.999), with the best sensitivity (100%) and specificity (75%) at a cut-off value of 1.7. They also stated that the cardiac SUV retention index performed similarly well (AUC: 0.999), while from the planar indices, the heart to contralateral lung ratio (H/CL) had the best AUC (0.987), but was lower compared to the SPECT parameters.

Wollenweber et al. [37] aimed to establish quantitative SPECT as a means to detect, treat and monitor cardiac ATTR. The authors evaluated myocardial SUV_peak_ and its values normalised to bone activity (nSUV_peak_) and soft tissue uptake (wSUV_peak_) and discovered that a SUV_peak_ cut-off of 3.1 could accurately differentiate Perugini score 2–3 from Perugini score 0–1 (sensitivity and specificity 100%).

Ren et al. [38] measured multiple SUV values in three groups of patients: a group with individuals suffering from cardiac ATTR, a group with patients diagnosed with cardiac AL and one with patients known to have other cardiac diseases. They discovered that SUV_max_, SUV_mean_ and SUV_median_ were significantly higher in the group suffering from cardiac ATTR than in the other two groups, in concordance with the observations made with the H/CL ratio measured on planar scintigraphy.

A more detailed study was proposed by Dorbala et al. [39], who analyzed SUV_max_, SUV_mean_, cardiac amyloid activity (CAA) and ID in 72 patients that had ^99m^Tc-pyrophosphate (^99m^Tc-PYP) SPECT/CT examinations performed in their centre. The authors applied receiving operator curves for all aforementioned parameters and discovered that all 4 quantitative metrics were highly accurate for the diagnosis of cardiac ATTR, being able to correctly differentiate between Perugini grades 0–1 and 2–3. They have also stated that out of the four parameters, SUV_mean_ was the least repeatable among the observers.

Ben-Haim et al. [40] evaluated a larger number of quantitative SPECT metrics, correlating cardiac uptake to bone, liver, lung and soft tissue uptake. The investigators observed that a SUV_max_ cut-off of 6.0 could accurately separate patients with cardiac ATTR from other non-ATTR individuals, with a false positive rate of 0% and true positive rate, positive and negative predictive values of 100%. The authors obtained the same results for SMaT20, SMaT40 and SMaT60 cut-offs of 2.5, 3.3 and 4.2, respectively. SMaT20,40,60 (cardiac)/SUV_mean_ liver and SMaT20,40,60/SUV_mean_ (liver/lung) were the best methods to separate between Perugini grades 2 and 3.

The largest patient cohort in which quantitative SPECT parameters were gathered was reported by Kessler et al. [41]. Their results revealed that SUV_max_ values showed a good linear relationship with Perugini score (*p* < 0.0001), with a cut-off of 6.1 presenting acceptable sensitivity and specificity (98.7% and, respectively, 87.2%) in differentiating cardiac ATTR from other forms of non-ATTR cardiac afflictions. The same linear relationship was seen between the Perugini score and myocardium-to-blood pool ratio and, respectively, between the Perugini score and myocardium-to-vertebrae ratio.

The latest study to evaluate the diagnostic potential of quantitative SPECT/CT was conceived by Avalon et al. [42], who examined the correlations between the semiquantitative planar indices and quantitative SPECT parameters in 77 patients who underwent ^99m^Tc-PYP scintigraphy. Patients were grouped into two cohorts based on their semiquantitative indices, and SPECT parameters, acquired at 1 h and 3 h postinjection, were compared between the two groups. The authors established that a cut-off value of 1.88 showed the best sensitivity and specificity in distinguishing between ATTR-positive and ATTR-negative patients. They also stated that SUV_max_ at 1 h correlated with Perugini score, H/CL ratio and CMR parameters.

**Figure 3:**
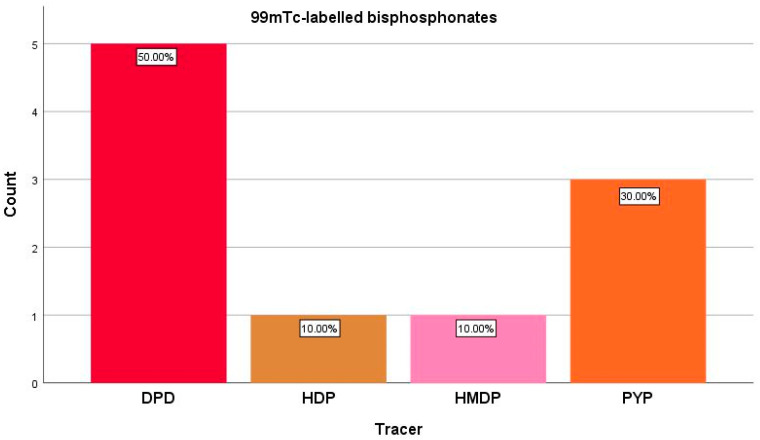
Radiotracers distribution among the included studies. Abbreviations: DPD: 3,3-diphosphono-1,2-propanodicarboxylic acid; HDP: hydroxy-methylene diphosphonate; HDMP: hydroxy-dimethylene diphosphonate; PYP: pyrophosphate.

All studies summed 554 patients who had their cardiac uptake of bone-avid radiotracers measured both on planar and SPECT/CT imaging. Most studies (50%) used ^99m^Tc-DPD as a radiotracer for cardiac ATTR evaluation, the second most used radiopharmaceutical being ^99m^Tc-PYP (30%), with only one study each (10%) for ^99m^Tc-HDP and ^99m^Tc-HDMP (Figure 3). The time interval from radiopharmaceutical injection to exam acquisition varied from 1 h, in Ren et al. [38] and Avalon et al. [42] studies, up to 4 h, in the research of Caobelli et al. [34], the most consistent uptake interval being 3 h [33,36,40,41]. Five studies mentioned performing studies on experimental phantoms for accurate calibration of the gamma camera [35,37,38,39,41].

The standard of references for cardiac uptake, used unanimously (100%), was the Perugini score, aided or not by other semiquantitative planar indices. Four studies, Scully et al. [36], Wollenweber et al. [37], Ren et al. [38] and Avalon et al. [42], included the heart-to-contralateral lung ratio (H/CL) in their analysis of the correlation of planar scintigraphy to SPECT/CT parameters, while Wollenweber et al. [37] and Ren et al. [38] also analyzed the connection of heart-to-whole body ratio (H/WB) to SPECT results.

**Figure 4:**
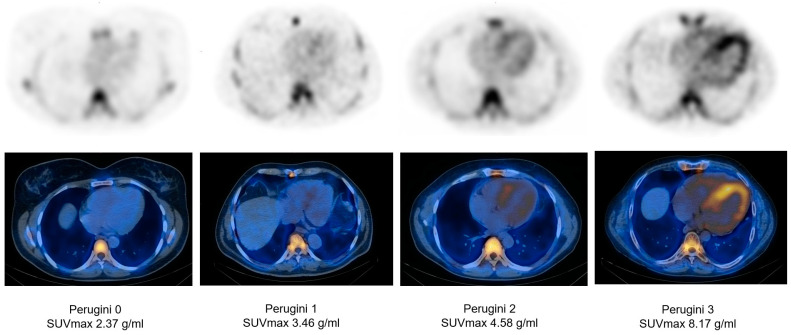
SPECT/CT-derived myocardial SUV_max_ variation in correlation to Perugini grading (images from our laboratory).

Regarding the quantitative SPECT/CT parameters chosen by each investigative group and considering the software used for quantitation, numerous parameters were measured throughout the studies, normalized or not to other healthy tissue uptake, with the most consistent being SUV_max_, analysed in 8 papers (Figure 4). The studies that did not analyse SUV_max_ were Scully et al. [36] and Wollenweber et al. [37], who chose to measure SUV_peak_ as their main SPECT parameter. Other frequent SPECT parameters used to describe cardiac uptake in cardiac ATTR were SUV_mean_ [38,39,42], ID [35,39] and cardiac SUV retention index [36,42].

## 4. Discussion

To our knowledge, this is the first systematic review to analyze the utility of quantitative SPECT/CT parameters in the diagnosis and surveillance of ATTR cardiomyopathy. Quantitative analysis of SPECT/CT data in cardiac amyloidosis has gained interest in recent years, mainly because of the imperative need for monitoring patients treated with newly developed medication, which possesses the potential of decreasing the amyloid burden.

The present expert consensus agrees that to enable early diagnosis of cardiac ATTR, evaluation of myocardial uptake on bone scintigraphy should be considered in patients with heart failure, unexplained neuropathy, family history of amyloidosis or unexplained increase in LV wall thickness on conventional imaging methods [43]. Bone scintigraphy facilitates the diagnosis of cardiac ATTR amyloidosis to be made reliably without the need for histopathological proof in patients who do not have a monoclonal gammopathy [44]. At present, appropriate evidence of cardiac ATTR on cardiac ultrasound or CMR, combined with the absence of light chain clones and Perugini visual score ≥ 2 myocardial uptake of ^99m^Tc-labelled bisphosphonates on planar scintigraphy, is diagnostic of ATTR cardiomyopathy, in which case endomyocardial biopsy is unnecessary [11,43,44]. Our review evaluates the diagnostic potential of quantitative SPECT/CT analysis in cardiac ATTR and its correlation to the already established semiquantitative and qualitative interpretation of planar bone scans. New advances in SPECT gamma cameras, added to the recent standardization of quantitative SPECT/CT, have opened the doors for new applications of SPECT-based parameters [28]. Five of the studies included in our review [35,37,38,39,41] mentioned performing phantom studies to assess the validity of their results. Bellevre et al. [35] and Dorbala et al. [39] used cardiac dedicated gamma cameras equipped with cadmium-zinc-telluride (CZT) collimators to acquire their planar and SPECT/CT scans. CZT-based gamma cameras have been demonstrated to be of greater quality for molecular imaging targeting the heart, especially in the case of myocardial flow and perfusion, and may present good prospects for other cardiac-related types of scintigraphy [45,46].

**Figure 5:**
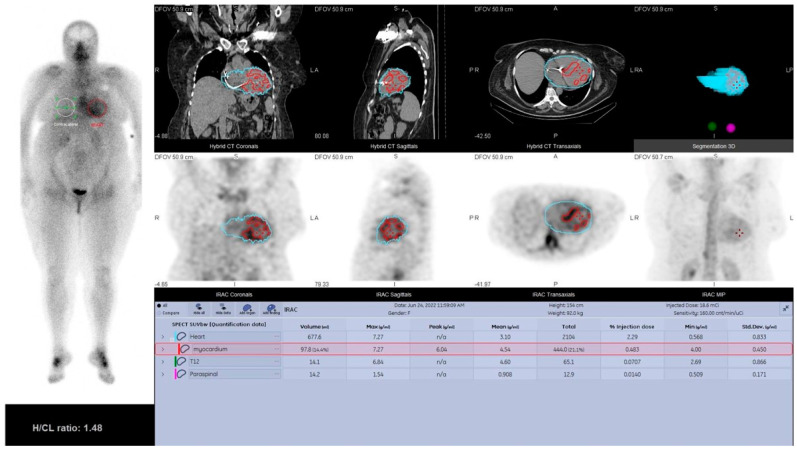
47 year old patient diagnosed with familial ATTR (Glu54Gln). The planar bone scan shows ^99m^Tc-PYP cardiac uptake greater than bone uptake, transcribing into Perugini score 3. Quantitative SPECT/CT confirms this observation, with the myocardium showing an SUV_max_ of 7.27 g/mL, which is higher than the one in bone (namely T12 vertebrae), with SUV_max_ of 6.84 g/mL (Images from our laboratory. Quantitative SPECT/CT was performed using Q.VolumetrixAI software, provided by General Electric Healthcare, on images obtained 3 h post-radiotracer administration. The heart volume was manually segmented and a region-of-interest for measuring the SUV_max_ of the myocardium was inserted and adjusted to the myocardial uptake).

The increased number of parameters analyzed by each investigative group, added to the heterogeneity of the patient cohorts and the usage of different software to execute the analysis, makes performing a meta-analysis inadequate. Furthermore, the lack of procedure standardization would not convey accurate and reproducible results, as the investigators presented different time intervals between the bone-specific radiotracer administration and the beginning of imaging acquisition, which would alter the SUV calculations given the ^99m^Tc decay scheme. However, a recent review published by Khor et al. [47] concludes that the optimal timepoint for performing SPECT/CT in patients evaluated for cardiac amyloidosis is 3 h post-radiotracer administration, to minimize the blood pool uptake that would lead to false positive results. We observed that SUV_max_ was the most used parameter among the studies, with only two studies, Scully et al. [36] and Wollenweber et al. [37], using SUV_pea_k as their main measured parameter. We noticed that other SUV-derived parameters were calculated in relation to the ^99m^Tc-labelled bisphosphonates uptake of other tissues, such as muscles, bones and blood pool, with the intention of normalizing the myocardial uptake to other organs that may or not be involved in ATTR (Figure 5).

There are still some issues on the utility of quantitative SPECT/CT in cardiac ATTR evaluation that need to be further addressed through more extensive studies. Although quantitative SPECT/CT proved to accurately distinguish the ATTR-positive patients, which correlate to Perugini 2–3 grades in the absence of monoclonal proteins, one major problem that still remains is the capability of SPECT-based parameters to determine the presence of TTR amyloid in grade 1 patients. As low uptake degrees of bone-seeking agents have been shown to be present in cardiac AL as well [38], and some ATTR variants, such as Phe64Leu [48], Glu61Ala [49] or Val50Met [50], are known to have minimal infiltration in the heart, grounding diagnosis solely on the SPECT-based parameters in grade 1 patients would be deceiving, if not hazardous. This statement is further reinforced by a recent extensive study by Rauf et al. [51], which investigated 183 patients with grade 1 disease and discovered that while 58% of individuals had AL, 41% of the patients were positive for ATTR, most of them with no special gene variant involved. Both the paper of Rauf et al. [51] and a recent review by Oerlemans et al. [52] reinforce the need for genetic sequencing and histological identification and typing of amyloid in patients that do not fulfil the non-biopsy diagnostic criteria of ATTR and who would benefit from an early diagnosis of the disease.

The scarcity of the literature evaluating quantitative SPECT/CT parameters may also represent an impediment when discussing its value in evaluating the treatment response and changes in amyloid burden imprinted by recently discovered drugs. Biological understanding of cardiac ATTR has led to effective targeted therapies that can either stabilize the already existing transthyretin or silence its production [53]. Unfortunately, none of the present studies analysed their quantitative SPECT/CT parameters in relation to patient treatment response; this may represent a perspective for future research. Bellevre et al. [54] monitored an ATTR patient treated with Tafamidis for six months and reported that although the visual Perugini score remained unchanged, a decrease in the values of the SPECT parameters was observed, suggesting that quantitative SPECT appears to be a promising tool in evaluating treatment response in this kind of patient.

Although bone-seeking tracers have developed primarily around ATTR, their diagnostic performance for other types of cardiac amyloidosis is low, thus raising the opportunity for targeted amyloid PET/CT. Studies have shown that PET/CT with amyloid-avid tracers has the ability to identify all amyloid deposits, regardless of the precursor protein [55]. ^18^F-labelled PET radiotracers tend to be more useful in the diagnosis of cardiac AL than ATTR, presenting persistent uptake on both early and late acquisitions, whereas radiotracer uptake decreases in late images in the case of ATTR [55,56]. A study by Genovesi et al. [57] reported that dynamic quantitative analysis, with calculation of SUV_mean_ from time-activity-curves (TAC), is more useful in assessing the cardiac amyloid burden on PET/CT, accurately differentiating AL from ATTR. Additional research by Cuddy et al. [58] evaluated the cardiac retention index of ^18^F-florbetapir as a parameter for measuring the amyloid burden in cardiac AL and stated that its values correlate with the findings observed with CMR. These results from cardiac PET offer the potential for treatment monitoring in patients diagnosed with cardiac AL as well, without diminishing the value possessed by SPECT/CT with bone-seeking tracers in the case of cardiac ATTR.

## 5. Conclusions

Despite the limited number of papers published in the literature so far, the use of quantitative SPECT/CT with ^99m^Tc-labelled bone-avid radiotracers offers good prospects in the assessment of transthyretin cardiac amyloidosis. Nevertheless, more studies that could lead to a standardization of the imaging procedure need to be performed to establish accurate cut-off values that could differentiate between a positive and a negative scan, as well as to assess response to the newly developed therapies.

## Figures and Tables

**Table 1:** Characteristics of the studies that have researched the quantitative parameters of SPECT/CT in the management of cardiac ATTR. Abbreviations: HDP: hydroxy-methylene diphosphonate; DPD: 3,3-diphosphono-1,2-propanodicarboxylic acid; HDMP: hydroxy-dimethylene diphosphonate; PYP: pyrophosphate; SUV: standardized uptake value; ID: percentage of injected dose; HLR: heart-to-lung ratio; MBR: muscle-to-bone ratio; CAA: cardiac amyloid activity; SMaT: SUV mean above treshold; MBR*: myocardium-to-bloodpool ratio; MVR: myocardium-to-vertebral bone ratio; H/CL: heart-to-contralateral lung ratio; H/WB: heart-to-whole body ratio; NA: not applicable.

Reference	No. Patients	^99m^Tc-Labelled Radiopharmaceutical	Time from Injection to SPECT	Phantom Study	Segmentation Software	SPECT/CT Parameters	Planar Indices
Ramsay et al. [33]	29	HDP	3 h	No	Syngo.via (Siemens)	SUV_max_	Perugini score
Caobelli et al. [34]	13	DPD	3.5–4 h	No	Syngo.via (Siemens)	SUV_max_, SUV_peak_, nSUV_max_, nSUV_peak_,	Perugini score
Bellevre et al. [35]	30	HMDP	167 ± 25 min	Yes	Q.Metrix (General Electric Healthcare)	SUV_max_, ID, HLR, MBR	Perugini score
Scully et al. [36]	100	DPD	3 h	No	Hybrid Recon (Hermes Medical Solutions)	SUV_peak_, Cardiac SUV retention index	Perugini score, H/WB, H/CL
Wollenweber et al. [37]	32	DPD	2.5 h	Yes	Hermes Hybrid 3D (Hermes Medical Solutions)	SUV_peak_, nSUV_peak_, wSUV_peak_	Perugini score, H/WB, H/CL
Ren et al. [38]	37	PYP	1 h	Yes	MyoFlowQ	SUV_max_, SUV_mean_, SUV_median_	Perugini score, H/CL
Dorbala et al. [39]	72	PYP	2.5–3 h	Yes	MIM	SUV_max_, SUV_mean_, CAA, ID	NA
Ben-Haim et al. [40]	28	DPD	3 h	No	Q.Volumetrix MI	SUV_max_, SMaT_20_, SMaT_40_, SMaT_60_	Perugini score
Kessler et al. [41]	136	DPD	3 h	Yes	Pmod v3.2	SUV_max_, MBR*, MVR	Perugini score
Avalon et al. [42]	77	PYP	1 h and 3 h	No	NA	SUV_max_, SUV_min,_ SUV_mean_, Cardiac SUV retention index	Perugini score, H/CL

## Data Availability

No new data were generated or analysed in support of this research.
